# An Electrochemical Cholesterol Biosensor Based on A CdTe/CdSe/ZnSe Quantum Dots—Poly (Propylene Imine) Dendrimer Nanocomposite Immobilisation Layer

**DOI:** 10.3390/s18103368

**Published:** 2018-10-09

**Authors:** Kefilwe Vanessa Mokwebo, Oluwatobi Samuel Oluwafemi, Omotayo Ademola Arotiba

**Affiliations:** 1 Department of Applied Chemistry, University of Johannesburg, P.O. Box 17011, Doornfontein 2028, Johannesburg, South Africa; kvmokwebo@gmail.com (K.V.M.); ooluwatobi@uj.ac.za (O.S.O.); 2 Centre for Nanomaterials Science Research, University of Johannesburg, Doornfontein 2028, Johannesburg, South Africa

**Keywords:** Cholesterol, electrochemical biosensors, quantum dots, poly (propylene imine) dendrimer, cholesterol oxidase

## Abstract

We report the preparation of poly (propylene imine) dendrimer (PPI) and CdTe/CdSe/ZnSe quantum dots (QDs) as a suitable platform for the development of an enzyme-based electrochemical cholesterol biosensor with enhanced analytical performance. The mercaptopropionic acid (MPA)-capped CdTe/CdSe/ZnSe QDs was synthesized in an aqueous phase and characterized using photoluminescence (PL) spectroscopy, ultraviolet-visible (UV-Vis) spectroscopy, transmission electron microscopy (TEM), X-ray power diffraction (XRD), energy dispersive X-ray (EDX) spectroscopy. The absorption and emission maxima of the QDs red shifted as the reaction time and shell growth increased, indicating the formation of CdTe/CdSe/ZnSe QDs. PPI was electrodeposited on a glassy carbon electrode followed by the deposition (by deep coating) attachment of the QDs onto the PPI dendrimer modified electrode using 1-Ethyl-3-(3-dimethylaminopropyl)-carbodiimide hydrochloride (EDC), and N-hydroxysuccinimide (NHS) as a coupling agent. The biosensor was prepared by incubating the PPI/QDs modified electrode into a solution of cholesterol oxidase (ChOx) for 6 h. The modified electrodes were characterized by voltammetry and impedance spectroscopy. Since efficient electron transfer process between the enzyme cholesterol oxidase (ChOx) and the PPI/QDs-modified electrode was achieved, the cholesterol biosensor (GCE/PPI/QDs/ChOx) was able to detect cholesterol in the range 0.1–10 mM with a detection limit (LOD) of 0.075 mM and sensitivity of 111.16 μA mM^−1^ cm^−2^. The biosensor was stable for over a month and had greater selectivity towards the cholesterol molecule.

## 1. Introduction

Cholesterol ((3*β*)-cholest-5-en-3-ol) is a fat-like organic biomolecule found in all cells and that is naturally produced by the liver and the intestines. It plays an important role in the production of vitamin D, steroid hormones, and bile acid which assist in lipid digestion and also in maintaining the cell membrane [1,2,3,4]. Most food such as fatty red meat, eggs, cream, burgers, and animal fats contain high levels of cholesterol, which can lead to increase in the blood cholesterol level when consumed [3,4,5]. As important as cholesterol is for the body, it may cause health problems when it exists at a concentration higher than 5.2 mM (200 mg·dL^−1^) [5,6,7,8]. A high level of cholesterol can cause atherosclerosis, which is the hardening and narrowing of the heart arteries leading to an increased risk of cardiovascular diseases (CVDs) such as hypertension, stroke and heart attack [9,10,11]. CVDs are amongst the top three diseases that cause mortality and affect mobility in South Africa and the rest of the world [9,10,12]. Therefore, early detection, prevention and effective management of cholesterol level are of importance to human health and in diagnosis. Techniques such as high-pressure liquid chromatography (HPLC), mass spectroscopy, calorimetry, spectrophotometry, fluorimetry, electrochemistry and polarography have been used to analyze cholesterols and many other small biomolecules [7,13,14,15,16,17]. Among these methods, electrochemical detection methods, which include biosensors, have shown high potential owing to their high sensitivity, specificity and stability, rapid response time, cost-effectiveness, use of small sample volumes, miniaturisability and their good compatibility with advanced micromachining technologies [16,18,19,20,21]. Biosensors are analytical devices that incorporate a bioreceptor onto an electrochemical transducer for the purpose of measuring the biorecognition event between the bioreceptor and the target analyte. Biosensors have been used in the detection of many other biologically relevant analytes such as glucose, urea, thrombin etc. Despite the success recorded by biosensors, there is still a quest for improvement through the use of immobilization matrix and immobilization chemistry that will provide a biocompatible environment with good orientation for the enzyme [19,22,23,24,25].

The integration of biomolecules with nanomaterials to develop various types of electrochemical biosensors with amplified signals has stimulated a lot of attention. In the case of biosensors for cholesterol, Umar et al., design a cholesterol biosensor based on magnetic α-Fe_2_O_3_ micro-pine shaped hierarchical nanostructures, co-immobilized with cholesterol oxidase (ChOx) with a sensitivity of 78.56 μA μM^−1^ cm^−2^, a detection limit of 0.08 mM and a linearity of 0.1–8.0 mM [2]. Lata and co-workers also reported the development of an amperometric cholesterol biosensor based on on gold nanocomposites. The resultant biosensor had a detection limit of 0.01 mM and used a low potential of 0.27 V. Their report also showed that the biosensor can be reused more than 45 times and is highly stable for 60 days [26]. In another development, Li et al. fabricated ChOx/CS-GR/GCE cholesterol biosensor by covalently immobilising ChOx on chitosan-graphene (CS-GR) nanocomposite-modified GCE [27]. The presence of the nanocomposite did not only increase the electron transfer, but also enhanced the amount of ChOx immobilised [27]. Functionalised graphene and graphene nanocomposites have also been used in the development of cholesterol biosensors [28,29]. 

Recently, dendrimers have been exploited as a matrix or immobilisation layer to provide a biocompatible environment for biomolecules in biosensors. This is due to their unique properties such as: Analogues to small enzymes in terms of their size, functional complexity and structural precision, biocompatibility, cationic nature and multiple attachment sites [25,30,31]. Dendrimers such as poly (propylene imine dendrimer) dendrimer (PPI) and poly (amido amine) have been used as immobilisation layer in different types of biosensors for DNA, toxins, cholera, cancer biomarker etc. [25,31,32,33,34,35,36,37]. 

Another group of nanomaterials of interest in biosensor design are quantum dots (QDs) [18,38,39]. Owing to their nanoscopic size similar to proteins, they can be used to improve the surface area of the electrode leading to increase in electrochemical response and bio-receptor loading [38,40,41,42,43]. QDs have found application in photoelectrochemical biosensors where their unique responses in the presence of light is explored [44,45,46,47]. 

In this work, poly (propylene imine) dendrimer (PPI) and CdTe/CdSe/ZnSe core-multishell quantum dots are used as nanocomposite platform for a cholesterol oxidase (ChOx) based biosensor. Biomimicry, an important philosophy in biosensor design, demands the use of biocompatible materials with low toxicity. This motivates the use of core-multishell CdTe/CdSe/ZnSe quantum dots (QDs). We have earlier demonstrated that this QDs have low toxicity and are biocompatible [48]. Furthermore, the QD is expected to improve enzyme loading while the dendrimer provides a platform for biocompatibility and immobilization chemistry suitable for the enzyme attachment. 

## 2. Experimental Details

### 2.1. Material

Cadmium acetate, potassium tellurite, zinc acetate, 3-mercaptopropionic acid (MPA), sodium borohydride, sodium hydroxide, selenium powder (Se), sodium sulphite, potassium ferrocyanide, potassium ferricyanide. Potassium chloride, 1-Ethyl-3-(3-dimethylaminopropyl)-carbodiimide hydrochloride (EDC), N-hydroxysuccinimide (NHS), phosphate buffer (PBS) components (K_2_HPO_4_, KH_2_PO_4_), cholesterol oxidase (36 U·mg^−1^) from Streptomyces species, Enzyme Commission Number 1.1.3.6, cholesterol 1 × 1 mL chloroform 10 mg·mL^−1^, and platinum wire (Pt) were all purchased from Sigma Aldrich (Johannesburg, South Africa). Generation 2 (G2) (propylene imine) dendrimer (777.3 g·mol^−1^) from SyMO-Chem (Netherlands) and glassy carbon electrode (GCE) and Ag/AgCl (3 M Cl^−^) electrode were both from BASi (West Lafayette, IN, USA). All chemicals were of analytical grade and used as purchased. Deionized water was used throughout the synthesis and biosensor design. Cholesterol solution (10 mM) was prepared in the following manner: Triton-X 100 (5 mL) and isopropanol (5 mL) were mixed and heated at 50 °C, then 0.193 g of cholesterol was slowly added to the solution until fully dissolved. Thereafter, 40 mL of 10 mM PBS (pH 7.2) was added with continuous stirring to produce 10 mM stock solution of cholesterol. Different concentrations of cholesterol were prepared by proper dilution of the stock solution using the ratio 1:1:8 triton-X 100: isopropanol: PBS.

### 2.2. Instrumentation

UV-Vis absorption spectra were recorded on a PerkinElmer Lambda 25 UV-Vis spectrophotometer in the 200–900 nm wavelength range at room temperature with a resolution of 1 nm and scanning speed of 480 nm/min. Shimadzu RF-6000 photoluminescence spectrophotometer was used to monitor the photoluminescence properties. X-ray diffraction (XRD) measurements were performed with X’Pert Philips X-ray diffractometer equipped with monochromatic Cu Kα (0.1540 nm). Scanning electron microscopy (SEM) measurements were carried out using TESCAN Vega TC instrument (VEGA 3 TESCAN software) SEM instrument coupled with energy dispersive X-ray (EDX) at a voltage of 8.0 kV. Transmission electron microscopy (TEM) (JEOL 2100 HRTEM 200 kV, Tokyo, Japan) was used to carry out TEM measurements. All electrochemical measurements were carried out using three electrodes system made of GCE (working electrode), Ag/AgCl (3 M Cl^−^) (reference electrode) and Pt wire (counter electrode) in probes such as PBS and [Fe(CN)_6_ ]^3−/4−^ (in 0.1 M KCl).

### 2.3. Synthesis of CdTe/CdSe/ZnSe QDs

**Scheme 1:**
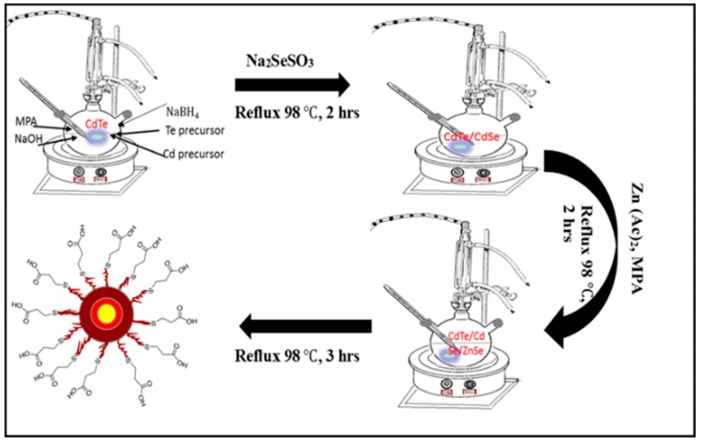
Schematic representation for the synthesis of CdTe/CdSe/ZnSe quantum dots.

CdTe/CdSe/ZnSe core/multishell QDs were synthesized using the reflux method reported by Ncapayi et al. [48] with slight modification. In a typical experiment, 0.051 g (0.2 mmol) of cadmium acetate dihydrate was dissolved in 50 mL of deionized water in a beaker containing a magnetic stirring bar. Then 17 µL (0.2 mmol) of 3-mercaptopropanoic acid (MPA) was added under continuous stirring. The pH of the Cd-MPA complex solution was adjusted from pH 3.67 to 12.01 using 1 M NaOH. The solution was then transferred into a 100 mL round bottom-three necked flask on a heating mantle and continuously stirred at a speed of 400 rpm. After 5 min of stirring, potassium tellurite (K_2_O_3_Te) solution prepared by dissolving 0.0103 g (0.04 mmol) K_2_O_3_Te in 50 mL deionized water was added to the Cd-MPA complex solution. The mixture was allowed to react for a further 5 min under continuous stirring, followed by addition of solid 0.08 g (2.0 mmol) NaBH_4_. This was also allowed to react for 5 min, followed by refluxing at 98 °C. The molar ratio of Cd:Te:MPA:NaBH_4_ was fixed at 1:0.2:1:10. Aliquots were taken at different time intervals to monitor the growth of CdTe core. After an hour of refluxing the above solution, 0.5 mL (1.8 mmol) sodium selenosulfate (Na_2_SeSO_3_) solution was swiftly injected into it. The solution was further refluxed for an hour while taking aliquots at different time intervals to monitor the formation of the CdTe/CdSe core/shell. The molar ratio of Cd:Te:Se was 1:0.2:9. In another flask, zinc (Zn) precursor solution was prepared by dissolving 0.043899 g (0.2 mmol) zinc acetate in 50 mL of deionized water followed by addition of 17 µL (0.2 mmol) mercaptopropionic acid (3-MPA) and pH optimization to pH 12.02 using 1M NaOH. After an hour of refluxing the above core/shell solution, Zn-MPA complex solution was swiftly added under continuous stirring. The molar ratio of Cd:Te:Se:Zn was 1:0.2:9:1. The prepared core/shell/shell was further stirred and refluxed for 7 h at 98 °C. Aliquots were taken at different time intervals to monitor the growth of CdTe/CdSe/ZnSe core/multi shell nanoparticles. Scheme 1 depicts the schematic representation involving the synthesis of the CdTe/CdSe/ZnSe QDs.

### 2.4. Fabrication of GCE/PPI/QDs/ChOx Biosensor

**Scheme 2:**
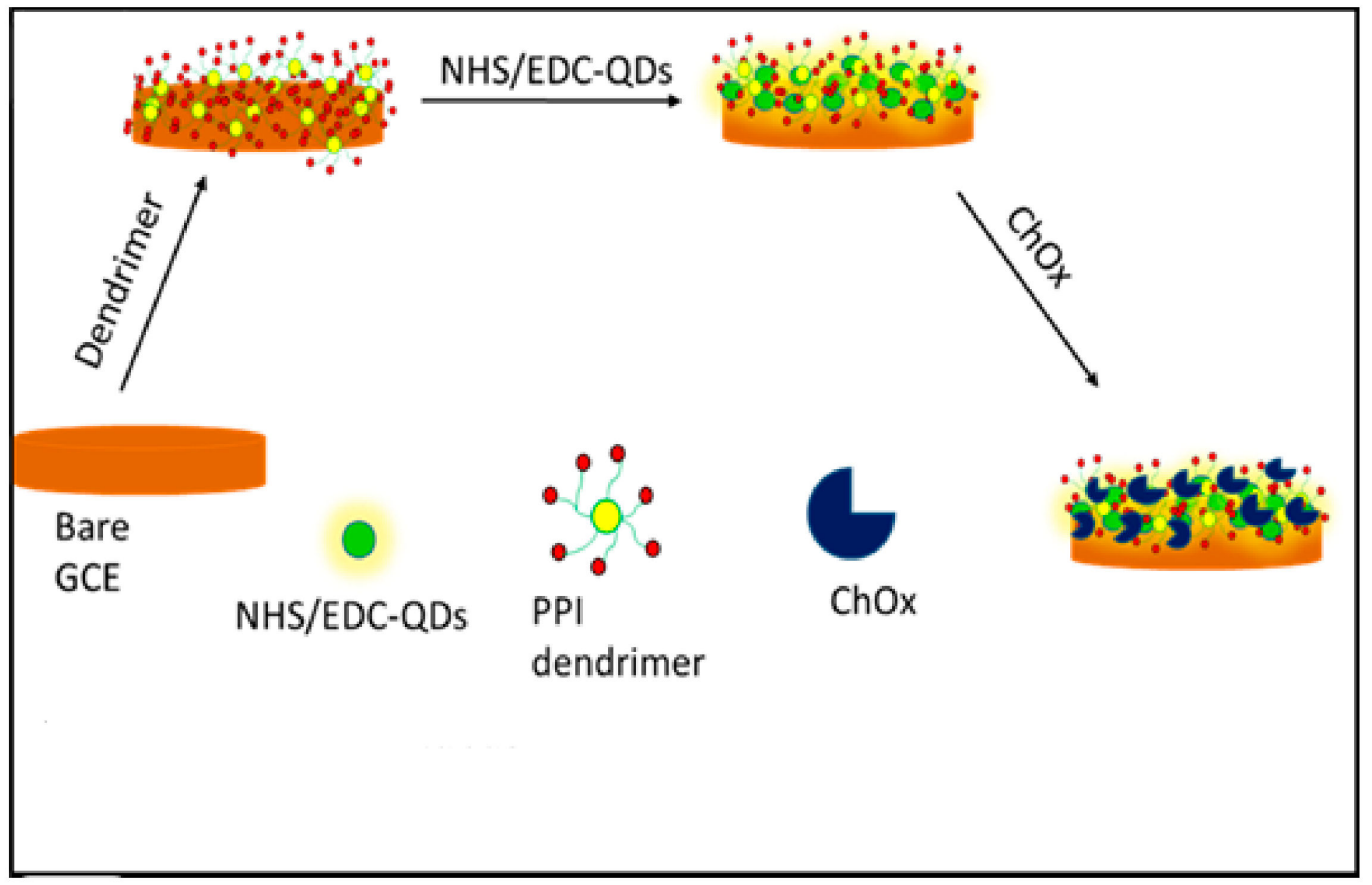
Schematic representation for the fabrication of GCE/PPI/QDs/ChOx biosensor.

Prior to electrode modification, the working glassy carbon electrode (GCE, 3 mm diameter) was cleaned consecutively with aqueous slurries of 1.0, 0.3 and 0.05 µm alumina powders, respectively on a micro-cloth pad and then rinsed thoroughly with double distilled water between each polishing step. The residual polishing materials on the electrode was removed by washing successively with a solution of ethanol and distilled water (1:1) in an ultrasonic bath, air-dried and then used immediately for measurements. The cleanliness of the electrode surface was confirmed by running cyclic voltammetry (CV) measurements in PBS solution (pH 7.2) from −0.3 V to 1.0 V at a scan rate of 50 mV·s^−1^. The GCE was modified with PPI by electrodepositing 10 mM PPI solution on the electrode surface using 10 cycles at 50 mV·s^−1^ from −0.5 V to 1.2 V. The electrode was left to dry at room temperature overnight and then washed with PBS to remove the unbound PPI. The PPI modified electrode (GCE/PPI) was modified with the as-synthesized QDs as follows: A 1 mL volume of MPA-capped QDs was first activated with 0.1 M EDC (200 μL) and 0.025 M NHS (200 μL) solution for 1 h with gentle stirring at room temperature. Then the GCE/PPI electrode was immersed into QDs solution and incubated for 2 h to allow the QDs to attach to the amine groups of the dendrimer through Schiff base method. The GCE/PPI/QDs electrode was allowed to dry at room temperature and then washed with PBS to remove any unbounded QDs. Finally, the GCE/PPI/QDs electrode was incubated in a solution of ChOx (2.0 mg·mL^−1^) for 6 h at 4 °C to allow the enzyme to immobilize on the electrode. The GCE/PPI/QDs/ChOx bio-nanoelectrode was allowed to dry and was used for the detection of cholesterol. The scheme that highlights the experimental protocol for the biosensor is depicted in Scheme 2. 

## 3. Results and Discussions

### 3.1. Characterization of CdTe/CdSe/ZnSe Quantum Dots

**Figure 1:**
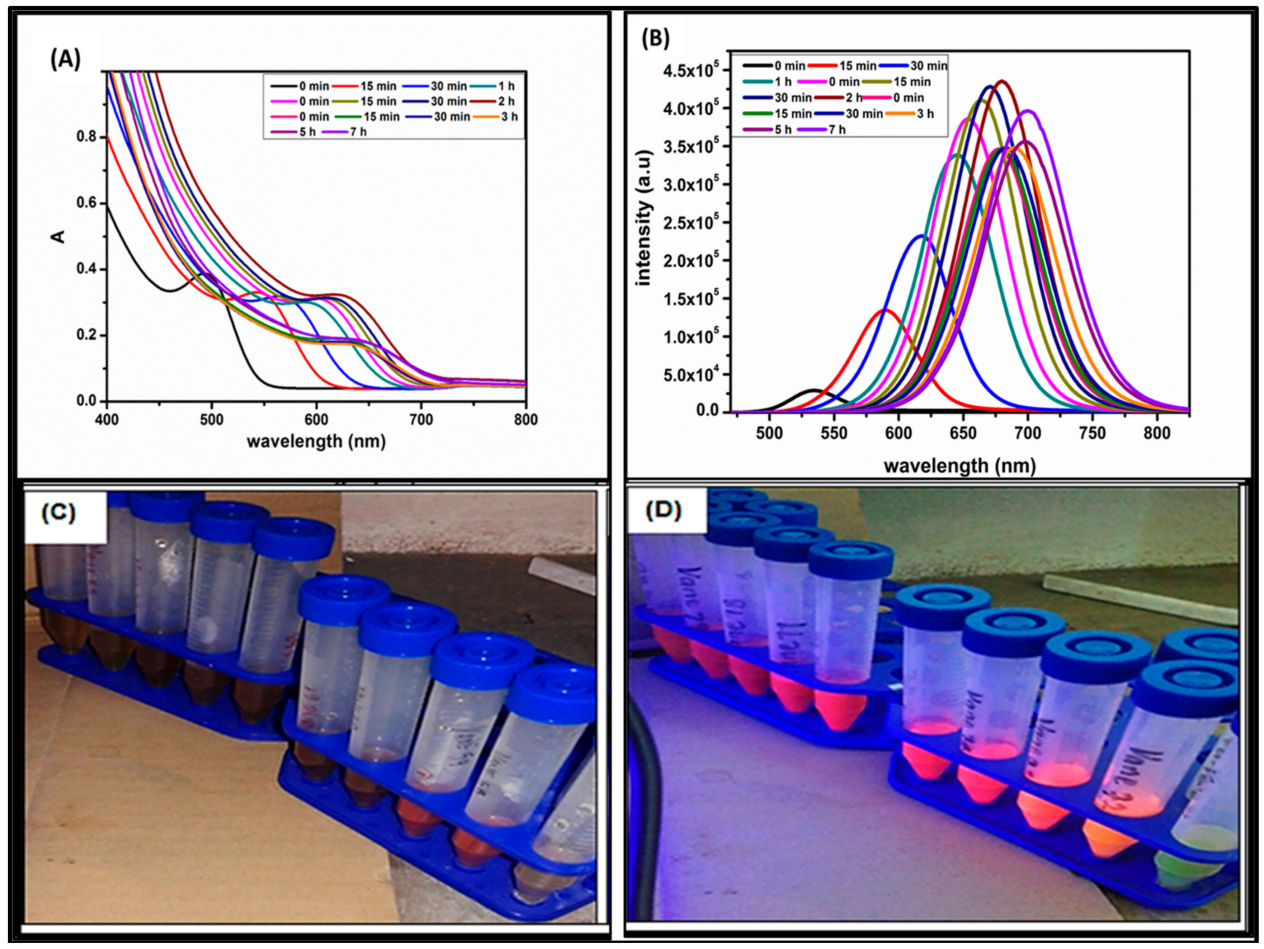
(**A**) UV-Vis absorption. (**B**) photoluminescence. (**C**) spectra of MPA-capped CdTe/CdSe/ZnSe core-multishell quantum dots at different growth stages and reaction time. Digital images of the as-synthesized CdTe/CdSe/ZnSe QDs (at different reaction time) under UV-lamp at an excitation wavelength of 250 nm. (**D**) 350 nm.

The optical properties of CdTe/CdSe/ZnSe core/shell/shell QDs at different growth stages and reaction time is shown in Figure 1A,B. All the nanoparticles emit from light yellow to brown (Figure 1C) and from yellow to deep red (Figure 1D) under UV light of 250 nm and 350 nm respectively. The average particle sizes of the QDs as calculated using Yu et al. method [49] ranges from 3.75–4.28 nm. The absorption wavelength of the CdTe core and CdTe/CdSe core/shell becomes red shift with an increase in absorbance and intensities as the reaction time increased (Figure 1A,B), indicating growth of the nanoparticles and shell formation [50]. The absorption spectra (Figure 1A) of the QDs show three stages of growth indicating the formation of three layers, which are CdTe core (502 to 610 nm, 0 min–1 h), CdTe/CdSe core/shell (602 to 626 nm, 0 min–2 h) and CdTe/CdSe/ZnSe core multishell (630 to 647 nm, 0 min–7 h). The significant red-shifts of about 24 nm and 17 nm, an hour after adding selenium and zinc precursors into the CdTe core and CdTe/CdSe core/shell solutions, respectively, confirm the formation of CdSe shell around the CdTe core (CdTe/CdSe) and ZnSe shell around the CdTe/CdSe core/shell (CdTe/CdSe/ZnSe) respectively. This red-shift shows that there is an increased leakage of excitons into the shells and formation of the core/shell and core/multishell QDs rather than formation of alloyed QDs [50,51,52]. The emission spectra (Figure 1B) of the QDs also show three stages of growth as reaction time increased and after the addition of both Se and Zn precursor respectively with band edge luminescence at an excitation wavelength of 450 nm. The narrowing of the emission peaks and high intensities are indicative of particles with focused size distribution [50]. This can be attributed to proper passivation by the shell formation via reduction of the electronic defects at the surface of the QDs [53,54]. The reduction in the absorption and PL intensities immediately after the addition of Zn precursor (during the formation of CdTe/CdSe/ZnSe QDs), can be attributed to the increase in the particle size, a high concentration of the unreacted Zn precursor and the formation of excess layers of ZnSe shell [48,52,55]. However, after 5 h of synthesis, the intensity of CdTe/CdSe/ZnSe core/shell/shell increased again. This increase is attributed to proper passivation of the QDs and depletion of excess precursor concentration as reaction time increased [50]. This following motivates for the synthesis of the CdTe/CdSe/ZnSe core mutlishell quantum dots: The ZnSe shell in CdTe/CdSe/ZnSe core multishell QDs was employed to provide perfect passivation of CdTe core and to reduce the toxicity of the cadmium based QDs via inhibiting the release of Cd^2+^ [56]. However, the lattice mismatch between CdTe and ZnSe is large and can cause strains between the CdTe core and ZnSe shell [50,56,57,58]. The defects caused by ZnSe shell have negative influences on both the photoluminescence quantum yield (PLQY) and the stability of the core/shell. Due to this lattice mismatch and defects, the CdTe core was first coated with CdSe shell because its band gap and lattice spacing lies at an intermediate level between those of the CdTe core and the ZnSe shell. This synthesis approach decreases toxicity, improves the PLQY and the photostability of the quantum dot.

**Figure 2:**
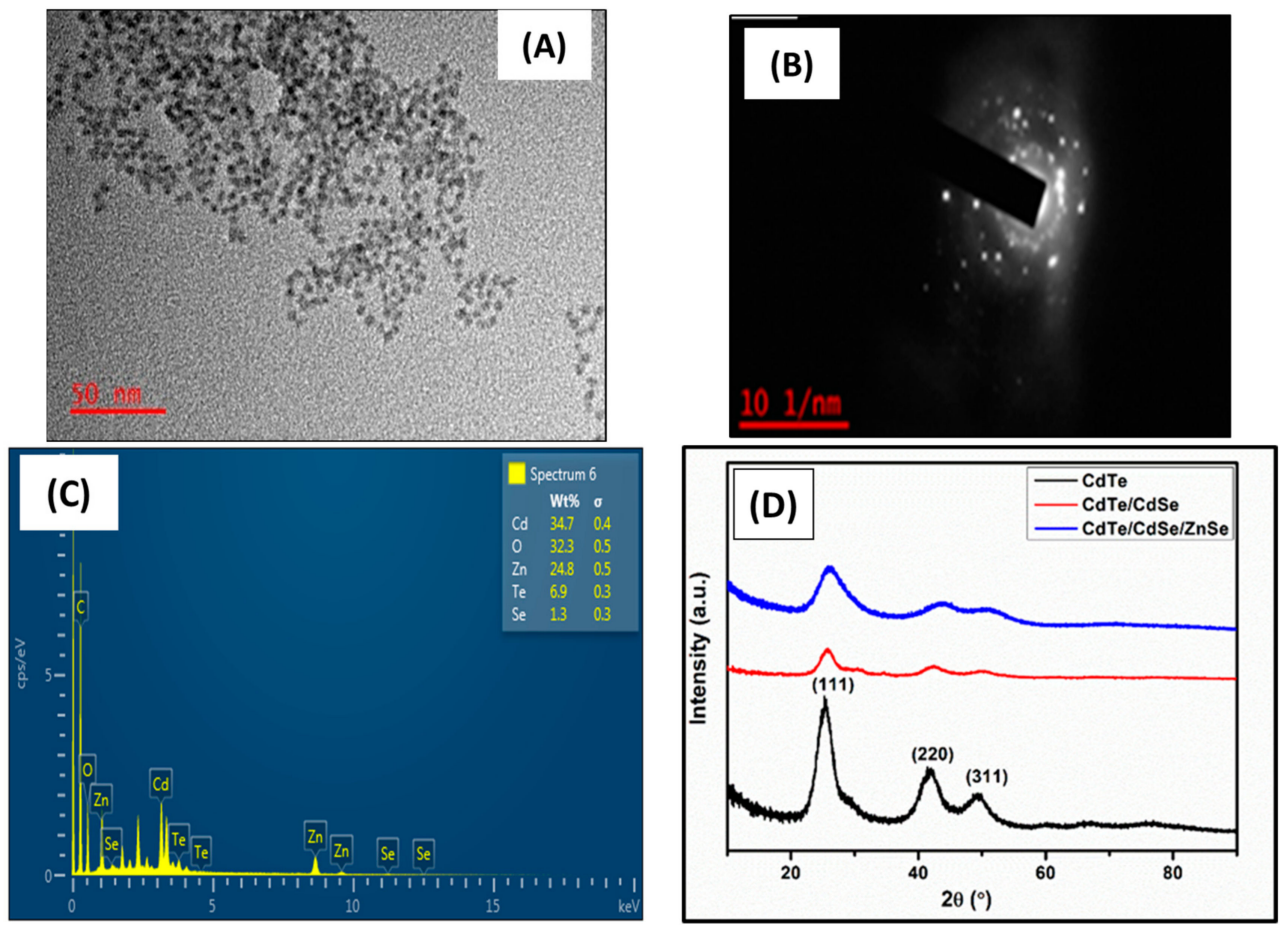
TEM image (**A**). SAED (**B**), EDX spectrum (**C**) and XRD spectra (**D**) of the as-synthesized MPA-capped CdTe/CdSe/ZnSe quantum dots.

The TEM image and corresponding selected area electron diffraction (SAED) patterns of the as-synthesized CdTe/CdSe/ZnSe QDs are shown in Figure 2A,B. The TEM micrograph (Figure 2A) shows that the as-synthesized materials are small, monodispersed and spherical in shape with an average particle size of 4.08 nm. The SAED pattern in Figure 2B shows a diffused rings pattern corresponding to (111), (220) and (311) reflections of a cubic crystal structure of Zn-blended phase. The elemental composition of the as-synthesized CdTe/CdSe/ZnSe QDs obtained using EDX (Figure 2C) confirms the presence of Cd, Te, Se, and Zn in the CdTe/CdSe/ZnSe QDs. The higher percentage of oxygen in the EDX spectrum of the QDs is due to the oxygen groups present in the capping agent, MPA. One is from the carbonyl (−C=O) group and the other from the hydroxyl (−OH) group. The crystallinity of the as-synthesized quantum dots was confirmed by XRD as as shown in Figure 2D. The XRD patterns of CdTe, CdTe/CdSe and CdTe/CdSe/ZnSe QDs consist of three diffraction peaks corresponding to (111), (220) and (311) cubic zinc blended structures [59]. For CdTe core, the diffraction peaks were located at 25.25°, 41.88° and 49.23° whereas for CdTe/CdSe and CdTe/CdSe/ZnSe the diffraction peaks were located at 25.73°, 42.38°, 50.38° and at 26.04°, 43.97° and 51.09°, respectively. After growing the CdSe and ZnSe shells on to the CdTe core, the diffraction peaks shifted to higher angles with broaden peak width. The shifting of the diffraction peaks to higher angles confirms the formation of the core/shell and core/shell/shell structure rather than an alloyed structure [60,61]. The average crystallite size of the CdTe/CdSe/ZnSe QDs as calculated using Scherrer’s equation is 4.32 nm—this agrees with TEM result. The stronger and narrower (111) peak in Figure 2D indicates that the nanocrystals were elongated along the c-axis. Furthermore, the broad nature of the XRD peaks is attributed to the nano-crystalline nature of the as-synthesized QDs, which is also consistent with the result from the optical spectroscopy.

### 3.2. Electrochemical Impedance Spectroscopy (EIS) Analysis

**Figure 3:**
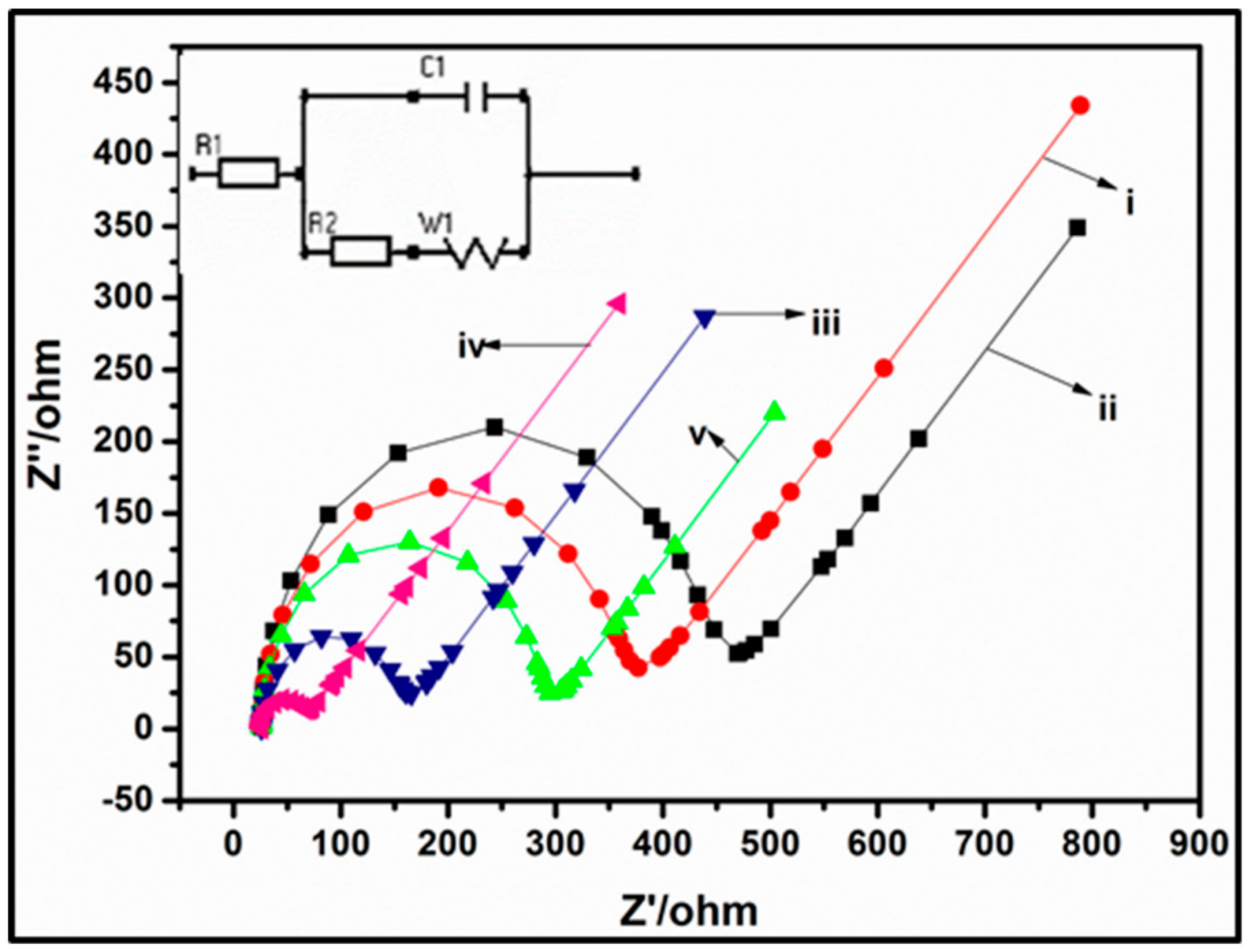
Nyquist plot of GCE (i); GCE/QDs (ii); GCE/PPI (iii), GCE/PPI/QDs (iv); and GCE/PPI/QDs/ChOx (v) in 10 mM [Fe (CN)_6_ ]^3−/4−^/0.1 M KCl redox probe in the frequency range of 10^3^ Hz to 1 Hz. Insert: Corresponding equivalent circuit obtained from the Nyquist plot.

To verify the successful fabrication of the bioelectrode (sensing platform), EIS was used to monitor the interfacial properties of the prepared electrodes after every modification step (Figure 3). The measurements were carried out at a potential of 0.2 V and a frequency 10^3^ Hz to 1 Hz using 10 mM [Fe(CN)_6_]^3−/4^] redox couple (1:1) with 0.1 M KCl as the supporting electrolyte. The value of the charge transfer resistance of the modified electrode was estimated by the semicircle diameter (charge transfer resistance, R_2_). Compared to bare GCE (Figure 3i), the diameter of the semicircle increased after modifying the GCE with MPA-capped CdTe/CdSe/ZnSe QDs (Figure 3ii) and this could be due to the electrostatic repulsion between the negatively charged [Fe(CN)_6_]^3−/4−^ redox couple and the negative charge on the MPA-capped QDs. A similar increase in EIS response after modification with an MPA capped ZnSe QDs in [Fe(CN)_6_]^3−/4−^ redox probe has been reported [62]. The change in R_2_ confirms that the QDs were successfully attached on the electrode surface. On the other hand, a reduction in R2 from 450 Ω (bare GCE) to 61 Ω (GCE/PPI) was observed after the electrodeposition of PPI on GCE (Figure 3iii and Table 1). This reduction suggests that PPI facilitated electron transfer rate at the interface of the electrode and [Fe(CN)_6_]^3−/4−^ owing it electrostatic attraction between the cationic PPI and the anionic redox probe [31,32,63]. Furthermore, the nanometric dimension of the PPI increased the electroactive surface area of the electrode for higher rate of electron transfer (reduced R_2_) [31,32,63]. The nanocomposite of the PPI and QDs on the GCE electrode resulted in further decrease in R_2_ (42 Ω). This marked reduction is a synergic effect resulting from the effect of the PPI and QDs. Firstly, the presence of both nanomaterials must have increased the surface area of the electrode than the individual nanomaterial. Secondly, the repulsion effect of the MPA capped QDs as observed in the R_2_ increase in Figure 3ii has been reduced markedly by the incorporation of the QDs into the PPI matrix. In the nanocomposite of PPI/QD, the negatively charged carboxylic group of the MPA capping agent has been linked to the amino group of the PPI using the crosslinking agents—EDC and NHS. The carbodiimide bond formed therefore reduced the negative charge density of the QDs thus negating the effect of repulsion (against FECN) observed in GCE/QDs. This counteracted repulsive force further enhanced the interfacial electron transfer leading to the observed R_2_ value of 42 Ω (Figure 3iv). A marked reduction in charge transfer resistance has also been observed through a synergic combination of PPI and gold nanoparticle [31]. When the GCE/PPI/QDs electrode was modified with 50 µL of ChOx (Figure 3v), an increase in electron transfer resistance was observed and this can be due to the poor conducting nature of the enzyme and also a possible electrostatic repulsion of the [Fe(CN)_6_]^3−/4−^ owing to the negatively charged enzyme. This increase in resistance shows that the enzyme was successfully immobilized on the platform. Table 1 shows the EIS circuit parameters obtained by fitting with Randle’s equivalent circuit.

### 3.3. Cyclic Voltammetric Behaviour of the GCE/PPI/QDs/ChOx Biosensor

**Figure 4:**
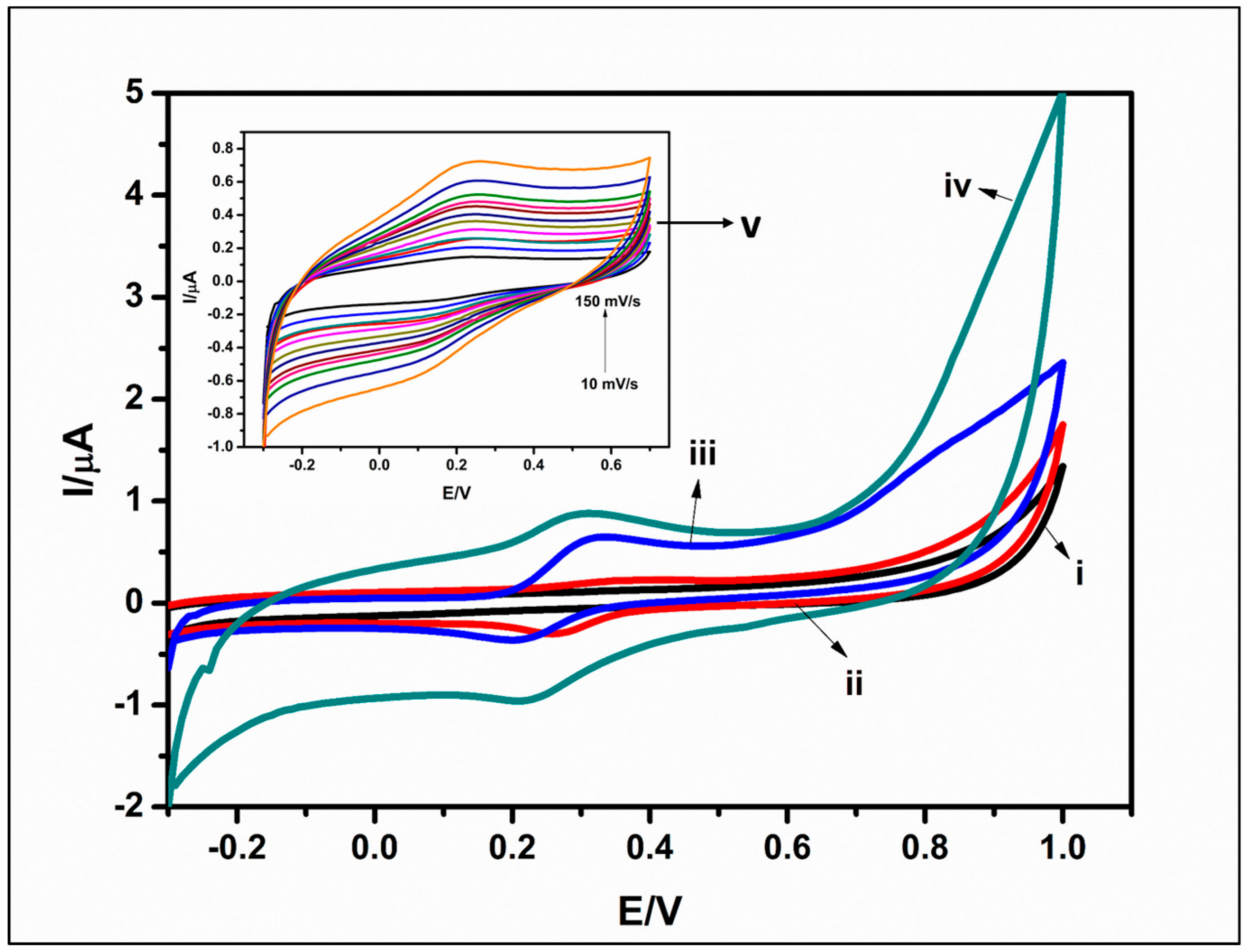
Cyclic voltammograms of GCE (i), GCE/QDs (ii), GCE/PPI (iii), GCE/PPI/QDs (iv), in 10 mM PBS (pH 7.2) at 50 mV.s^−1^. Insert: PPI/QDs/ChOx (v) in 10 mM PBS (pH 7.2) at different scan rates (10–150 mV.s^−1^).

The cyclic voltammetric behavior of each step of the biosensor development was investigated in 10 mM PBS (pH 7.2) at 50 mV·s^−1^. For the bare GCE (Figure 4i), as expected, no redox peaks were observed because there was no electroactive species on the electrode. The presence of CdTe/CdSe/ZnSe QDs on the electrode resulted in a small irreversible cathodic peak, which may be attributed to an electrochemical reaction on the electrode as a result of the presence of the QDs (Figure 4ii). For the dendrimer modified electrode (Figure 4iii), i.e., the GCE/PPI, a redox pair emerges at 0.295 V and 0.213 V (versus Ag/AgCl (3 M Cl^−^)). This redox couples can be attributed to the presence of the PPI dendrimer on the electrode surface. Similar electrochemical signatures have been reported by Arotiba [24,31,32] after the electrodeposition of PPI on carbon electrodes. The presence of QDs on the GCE/PPI/QDs (Figure 4iv) amplified the current observed in the redox couple attributed to PPI earlier (Figure 4iii). This amplification is a favorable property that may enhance the performance of the biosensor. It is interesting to note that after modification of the GCE/PPI/QDs with the enzyme, the redox peak due to the dendrimer and QDs was not quenched (Figure 4v). This shows that the PPI/QDs platform is able to act as molecular wire that permits redox signal and thus a suitable platform for the immobilization of the ChOx. Figure 4v inset resulted from scan rate study of the GCE/PPI/QDs/ChOx. It can be observed that there was no apparent shifting of the potential of the anodic or cathodic peak currents. This phenomenon can be used to infer the stability of the enzyme on the GCE/PPI/QDs platform. Again, this shows the suitability of the platform as an immobilization layer in the biosensor development.

### 3.4. Optimization Studies of the Designed Biosensor

**Figure 5:**
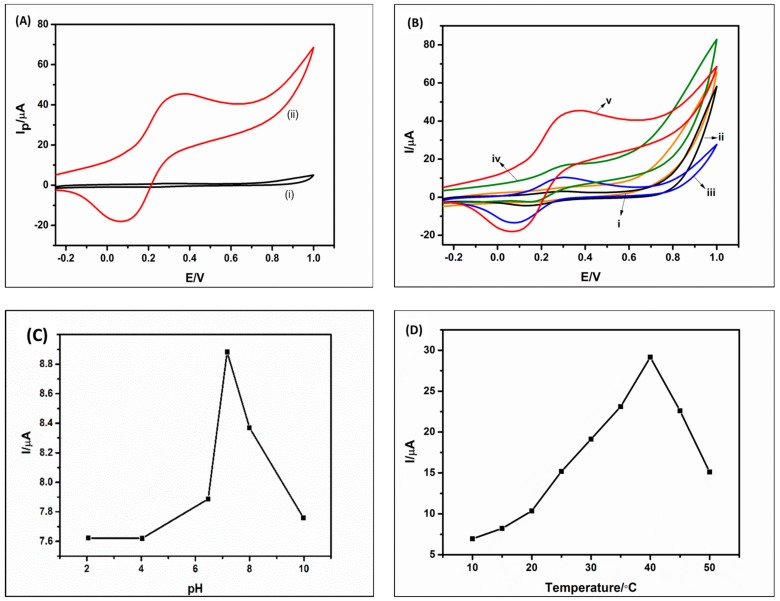
Cyclic voltammograms of GCE/PPI/QDs/ChOx in PBS (i) and in 10 mM cholesterol solution (ii) (**A**); Cyclic voltammograms of GCE/QDs (i), GCE/PPI (ii), GCE/PPI/ChOx (iii) GCE/QDs/ChOx (iv) and GCE/PPI/QDs/ChOx (v) in 10 mM cholesterol solution at 50 mV.s^−1^ (**B**); Effect of pH (**C**); and temperature (**D**) on the developed biosensor.3.5. Electrochemical Detection of Cholesterol Solution.

Figure 5 shows the optimization studies carried out on the biosensor. Figure 5A shows the response of the GCE/PPI/QDs/ChOx biosensor in PBS (i) and in PBS + Cholesterol (ii). The biosensor made of GCE/PPI/QDs/ChOx was able to detect the cholesterol in the solution and this is shown by the higher current redox peaks in Figure 5Aii. In the absence of the analyte, the response was poor and negligible (Figure 5Ai). CV was also used to study the effect of different modifiers on the electrocatalytic reaction between the biosensor and 10 mM cholesterol solution. The GCE modified with QDs (Figure 5Bi) and PPI (Figure 5Bii) did not show any significant redox current because there was no electrocatalytic reaction with the cholesterol due to the absence of the enzyme (ChOx). However, when ChOx was immobilized on the electrodes (Figure 5Biii,iv), some redox peaks were observed. Obviously, these peaks are from the enzymatic catalytic oxidation of cholesterol in the solution. The GCE/PPI/ChOx electrode shows a higher redox peak than that of GCE/QDs/ChOx. This suggests the biocompatible nature of dendrimer and a better immobilization ability than QDs. This is expected for the following reasons: 1. Electrostatic attraction between the positively charged PPI and the negatively charged enzyme. 2. The presence of amino groups on the PPI which could interact with the acidic group of the enzyme. 3. The nano-voids and opposite charge would have facilitated enzyme entrapment, host-guest chemistry or supramolecular chemistry [25]. Figure 5Bv, shows the highest redox current. This must have resulted from a synergic combination of PPI and QDs which enhanced the ChOx catalytic reduction of the cholesterol. Furthermore, the presence of QDs must have enhanced the enzyme wiring, thereby facilitating the exchange or flow of electron from the redox center of ChOx to the analyte (Cholesterol). The pH and temperature of PBS strongly affected the electrocatalytic activity of the developed biosensor. The effect of pH on the biosensor response was studied over a pH range of 2.0–10.0. Figure 5C shows that the peak current increased from pH 4.0 to pH 7.2 and then gradually decreased until pH 10.0. The decrease in the peak current after pH 7.2 can be attributed to poor enzymatic activity in basic medium. Therefore, the optimum pH used was 7.2 as expected. Figure 5D shows the effect of temperature on the developed biosensor (10–50 °C). Enzymes tend to denature and lose their bioactivity at temperature higher than 37 °C. However, for this work, a temperature of 40 °C was optimum.

**Figure 6:**
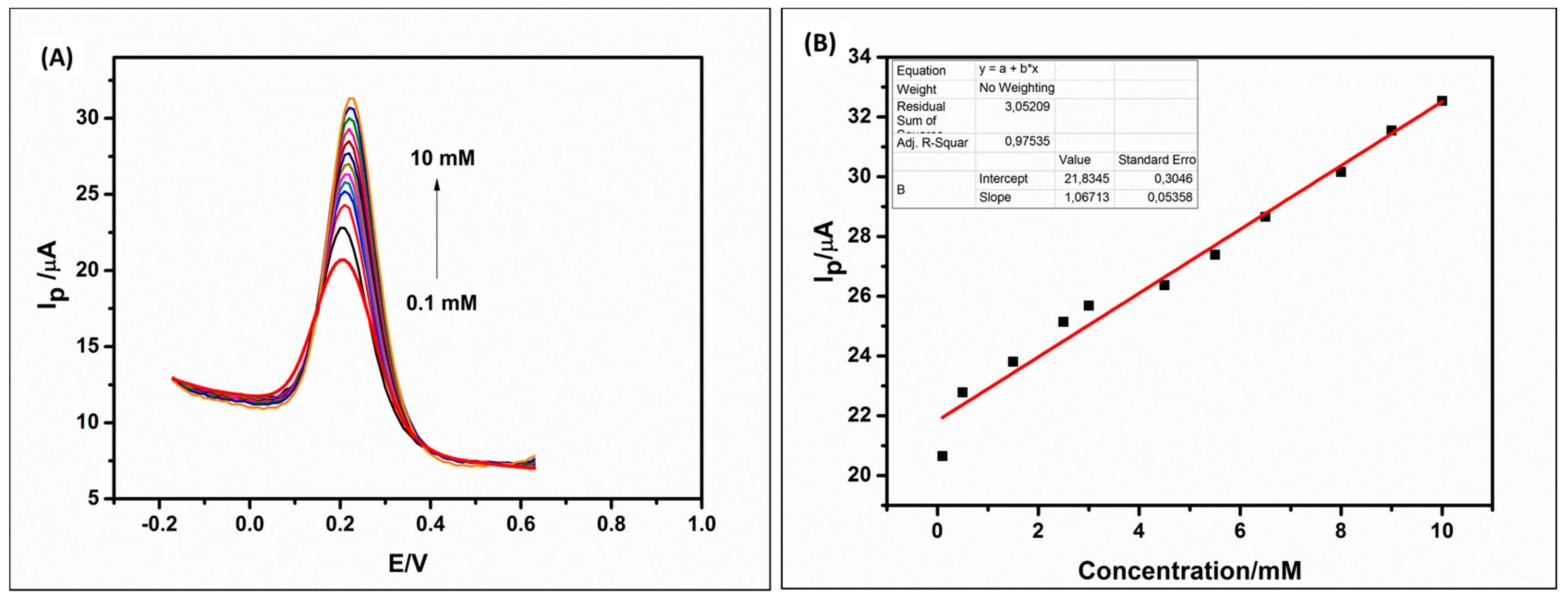
Square wave voltammetry (SWV) of GCE/PPI/QDs/ChOx biosensor upon successive addition of cholesterol (0.1–10 mM) in 10 mM PBS (pH 7.2) (**A**). Calibration plot of cholesterol at different concentrations (**B**).

After optimization, the biosensor was used to detect different concentrations of cholesterol standards using square wave voltammetry (SWV). Figure 6 shows the linear relationship between the current response and cholesterol concentrations during the bio-recognition process. When cholesterol is oxidized in the presence of the enzyme cholesterol oxidase and oxygen, it produces cholestenone and hydrogen peroxide (H_2_O_2_) [64,65]. The produced H_2_O_2_ is further reduced at the electrode surface producing electrons which increase the current of the solution being detected by the electrode (biosensor). According to stoichiometric ratios in Equations (1) and (2), one can conclude that the concentration of the analyte (cholesterol) is directly proportional to the hydrogen peroxide (H_2_O_2_) produced and also to the current produced [65]. Therefore, the higher the concentration, the higher the peak current produced.

(1) $$\mathrm{Cholesterol}\;+\;O_{2}\overset{ChOx}{\to }\mathrm{Cholestenone}\;+\;H_{2}O_{2}$$

(2) $$H_{2}O_{2}\;\to \;2H^{+}\;+\;O_{2}\;+\;2e^{-}$$

Prior to the detection of cholesterol, the initial blank was run 8 times using the GCE/PPI/QDs/ChOx bioelectrode so as to obtain a uniform steady current and then calculate the standard deviation which was used in determining the limit of detection (LOD). In Figure 6, the SWV plot and the corresponding calibration curve shows that the current of the biosensor increases steadily as cholesterol was added to the 10 mM PBS solution from 0.1 to 10 mM. The calibration plot shows a linear relationship from 0.5 to 10 mM with a correlation coefficient (R^2^) of 0.9754, LOD of 0.075 mM and sensitivity of 111.16 μA·mM^−1^ cm^−2^. The limit of detection was determining using the formula LOD = 3(S/m), where S is the standard deviation of the blank (PBS) before addition of cholesterol and m is the slope obtained from the calibration curve. The sensitivity was obtained by dividing the slope of the calibration curve by the active surface area of the GCE/PPI/QDs/ChOx biosensor. The analytical performance of the developed biosensor compared to those reported for cholesterol is shown in Table 2. Though reports on lower detection limit are in the literature (Table 2), the biosensor developed here still has a clinically relevant detection limit [52,53]. Furthermore, the method reported herein has a wide dynamic range with good stability. We have also demonstrated its selectivity such as glucose and ascorbic acid. The novel concept of dendrimer—QDs can form the basis for the preparation of other biosensors.

### 3.5. Selectivity, Stability and Reproducibility of Cholesterol Biosensor

**Figure 7:**
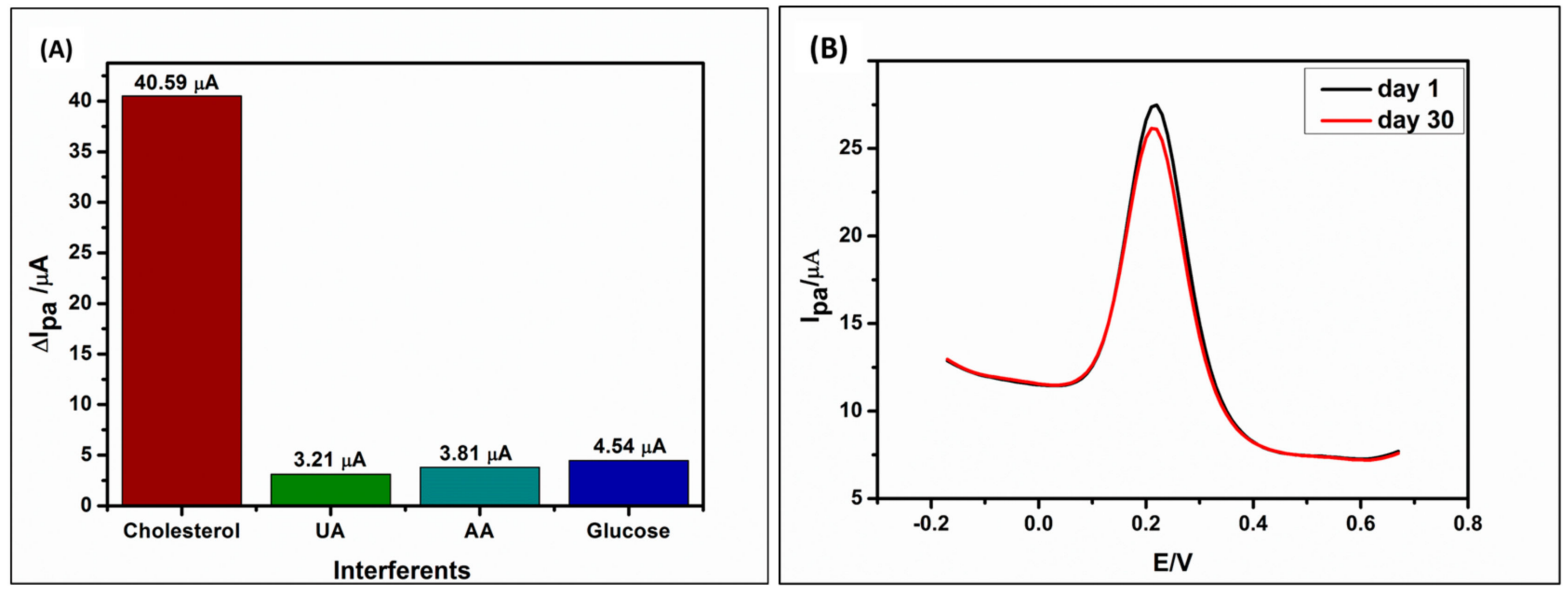
Selectivity of the biosensor (GCE/PPI/QDs/ChOx) in the presence of 5 mM cholesterol and interferents—10 mM uric acid (UA), 10 mM ascorbic acid (AA) and 10 mM glucose in PBS (pH 7.2) solution (**A**). Stability studies of the biosensor at day 1 and day 30 in 5 mM cholesterol solution at pH 7.2 (**B**).

The selectivity of the biosensor was evaluated in the presence of interferents, such as glucose (Glu), ascorbic acid (AA) and uric acid (UA) which co-exist with cholesterol in the serum. The effect of interferences was determined by adding the interference species (e.g., Glu) in the reaction mixture containing cholesterol (Figure 7A). The response of these interferents at the prepared electrode were 6.16%, 7.31% and 8.71% for UA, AA and Glu respectively. For cholesterol, the current produced was about 78% high than the interferents. Thus, the biosensor (GCE/PPI/QDs/ChOx) shows good selectivity and therefore has the potential to be used in real samples. For stability study, the biosensor was stored in the refrigerator at 4 °C when not in use and cleaned using 10 mM PBS (pH 7.2) before utilization. The stability of the working electrode was investigated for over four weeks in 5 mM cholesterol at 25 °C. After a month of storage at 4 °C, the biosensor retained 97% of its original response in the same sample, indicating that the biosensor had good stability when stored correctly (Figure 7B).

## 4. Conclusions

Highly fluorescent water-soluble MPA-capped CdTe/CdSe/ZnSe core/multishell quantum dots were successfully synthesized in aqueous phase and characterized using various analytical techniques. UV-vis, PL and XRD confirmed the growth and the core-multishell properties of the as-synthesized QDs. The as-synthesized CdTe/CdSe/ZnSe QDs and PPI nanocomposite was used as a platform for the development of GCE/PPI/QDs/ChOx-based cholesterol biosensor. The nanoplatform exhibited reversible electrochemistry, excellent catalytic properties towards [Fe(CN)_6_]^3−/4−^ probe and good electron transportation. The biosensor performance was greatly improved on the GCE/PPI/QDs/ChOx nanoplatform than in GCE/PPI/ChOx or GCE/QDs/ChOx nanoplatform alone. The biosensor had good sensitivity and selectivity, detection limit of 0.075 mM and a dynamic linearity of 0.1 mM–10 mM for cholesterol. Thus, this novel quantum dot-dendrimer platform lends itself to applications in other types of enzyme-based biosensors.

## Figures and Tables

**Table 1:** Impedance parameters obtained from circuit fitting of Nyquist plots (Figure 3) during electrode modification and biosensor design. R_1_ = solution resistance, R_2_ = charge transfer resistance, CPE = constant phase element, W_s_ = Warburg impedance.

Electrode Material	R_1_ (Ω)	R_2_ (Ω)	CPE (nF)	W_s_ (Ω)
Bare GCE	124	450	136	326
GCE/QDs	110	658	140	333
GCE/PPI	101	61	43	314
GCE/PPI/QDs	98	42	49	283
GCE/PPI/QDs/ChOx	103	256	112	321

**Table 2:** Comparison of analytical performance of some cholesterol biosensors constructed based on different modified electrode materials.

Electrode Material	Linear Range	Detection Limit	Sensitivity	Ref.
Nafion/ChOx/α-Fe_2_O_3_/Ag	0.1–8.0 mM	0.018 mM	78.56 μA μM^−1^ cm^−2^	[2]
ChOx-SCCPPy-g-C_3_NH_4_^+^/GCE	0.02–5.0 mM	8.0 μM	645.7 μA mM^−1^ cm^−2^	[66]
ZnO/ChOx	0.2–15.6 mM	0.4 mM	99.8 μA mM^−1^ cm^−2^	[67]
Si/Ag/ZNT/ChOx	1.0 μM–13.0 mM	0.5 nM	79.40 μA mM^−1^ cm^−2^	[68]
ChOx/PANI/PVP/Graphene	50 μM–10 mM	1.0 μM	34.77 μA μM^−1^ cm^−2^	[69]
ChOx/Chit./ZnO@ZnS/GCE	0.4–4.0 mM	0.02 mM	52.67 mA M^−1^ cm^−2^	[70]
ChOx-PB-modified SPE	0.05–0.30 mM	3.7 μM	26.0 mA M^−1^ cm^−2^	[71]
GCE/PTH/ChOx/HRP	0.025–0.125 mM	6.3 μM	0.18 μA μM^−1^ cm^−2^	[72]
GCE/PPI/QDs/ChOx	0.1–10 mM	0.075 mM	111.16 μA mM^−1^ cm^−2^	This work

## References

[1] Rahman M.M., Asiri A.M. (2015). One-step electrochemical detection of cholesterol in the presence of suitable K_3_Fe(CN)_6_/phosphate buffer mediator by an electrochemical approach. Talanta.

[2] Umar A., Ahmad R., Hwang S., Kim S., Al-Hajry A., Hahn Y. (2014). Development of highly sensitive and selective cholesterol biosensor based on cholesterol oxidase co-immobilized with *α*-Fe_2_O_3_ micro-pine shaped hierarchical structures. Electrochim. Acta.

[3] Batra N., Tomar M., Gupta V. (2015). ZnO-CuO composite matrix based reagentless biosensor for detection of total cholesterol. Biosens. Bioelectron..

[4] Gholivand M.B., Khodadadian M. (2014). Amperometric cholesterol biosensor based on the direct electrochemistry of cholesterol oxidase and catalase on a graphene/ionic liquid-modified glassy carbon electrode. Biosens. Bioelectron..

[5] De Ferranti S.D., Rodday A.M., Parsons S.K., Cull W.L., O’connor K.G., Daniels S.R., Leslie L.K. (2017). Cholesterol screening and treatment practices and preferences: A survey of United States pediatricians. J. Pediatr..

[6] Tığ G.A., Zeybek D.K. (2016). Fabrication of amperometric cholesterol biosensor based on SnO_2_ nanoparticles and Nafion-modified carbon paste electrode. Chem. Papers.

[7] Sharma D., Lee J., Seo J., Shin H. (2017). Development of a sensitive electrochemical enzymatic reaction-based cholesterol biosensor using nano-sized carbon interdigitated electrodes decorated with gold nanoparticles. Sensors.

[8] Jesch E.D., Carr T.P. (2017). Food ingredients that inhibit cholesterol absorption. Prev. Nutr. Food Sci..

[9] Norman R., Bradshaw D., Schneider M., Joubert J., Groenewald P., Lewin S., Steyn K., Vos T., Laubscher R., Nannan N. (2007). A comparative risk assessment for South Africa in 2000: Towards promoting health and preventing disease. S. Afr. Med. J..

[10] Expert Panel on Detection (2001). Executive summary of the Third Report of the National Cholesterol Education Program (NCEP) expert panel on detection, evaluation, and treatment of high blood cholesterol in adults (Adult Treatment Panel III). JAMA.

[11] Rosiek A., Leksowski K. (2016). The risk factors and prevention of cardiovascular disease: The importance of electrocardiogram in the diagnosis and treatment of acute coronary syndrome. Ther. Clin. Risk Manag..

[12] Wang J., Tan G.J., Han L.N., Bai Y.Y., He M., Liu H.B. (2017). Novel biomarkers for cardiovascular risk prediction. J. Geriatr. Cardiol..

[13] Pabbi M., Mittal S.K. (2017). An electrochemical algal biosensor based on silica coated ZnO quantum dots for selective determination of acephate. Anal. Methods.

[14] Qureshi R.N., Kaal E., Janssen H.-G., Schoenmakers P.J., Kok W.T. (2011). Determination of cholesterol and triglycerides in serum lipoproteins using flow field-flow fractionation coupled to gas chromatography–mass spectrometry. Anal. Chim. Acta.

[15] Yildiz H.B., Talaz O., Kamaci M., Caliskan A., Caliskan S. (2013). Novel photoelectrochemical biosensors for cholesterol biosensing by photonic “wiring” of cholesterol oxidase. J. Macromol. Sci. A.

[16] John J., Reghuwanshi A., Aravind U.K., Aravindakumar C. (2015). Development and validation of a high-performance thin layer chromatography method for the determination of cholesterol concentration. J. Food. Drug. Anal..

[17] Wu S., Wang Y., Mao H., Wang C., Xia L., Zhang Y., Ge H., Song X.M. (2016). Direct electrochemistry of cholesterol oxidase and biosensing of cholesterol based on PSS/polymeric ionic liquid—Graphene nanocomposite. RSC Adv..

[18] Kim K.-E., Kim T.G., Sung Y.-M. (2012). Fluorescent cholesterol sensing using enzyme-modified CdSe/ZnS quantum dots. J. Nanopart. Res..

[19] Lian K., Zhang P., Wang W., Dai T., Li L. (2014). Determination of total cholesterol in serum by gas chromatography-mass spectrometry. Asian J. Chem..

[20] Šabović I., De Toni L., Tescari S., De Filippis V., Menegazzo M. (2017). Detection of cholesterol and its oxidized derivatives in human sperm membranes through a fast and reliable LC-MS method. J. Clin. Lab. Med..

[21] Zhang X., Wei M., Lv B., Liu Y., Liu X., Wei W. (2016). Sensitive colorimetric detection of glucose and cholesterol by using Au@Ag core—Shell nanoparticles. RSC Adv..

[22] Grieshaber D., MacKenzie R., Voeroes J., Reimhult E. (2008). Electrochemical biosensors-sensor principles and architectures. Sensors.

[23] John S.V., Rotherham L.S., Khati M., Mamba B.B., Arotiba O.A. (2014). Towards HIV detection: Novel poly (propylene imine) dendrimer-streptavidin platform for electrochemical DNA and gp120 aptamer biosensors. Int. J. Electrochem. Sci..

[24] Shukla S.K., Mishra A.K., Mamba B.B., Arotiba O.A. (2014). Zirconia-poly (propylene imine) dendrimer nanocomposite based electrochemical urea biosensor. Enzyme. Microb. Technol..

[25] Soda N., Arotiba O. (2017). A polyamidoamine dendrimer-streptavidin supramolecular architecture for biosensor development. Bioelectrochemistry.

[26] Lata K., Dhull V., Hooda V. (2016). Fabrication and optimization of ChE/ChO/HRP-AuNPs/c-MWCNTs based silver electrode for determining total cholesterol in serum. Biochem. Res. Int..

[27] Li Z., Xie C., Wang J., Meng A., Zhang F. (2015). Direct electrochemistry of cholesterol oxidase immobilized on chitosan–graphene and cholesterol sensing. Sens. Actuat. B-Chem..

[28] Dey R.S., Raj C.R. (2010). Development of an amperometric cholesterol biosensor based on graphene—Pt nanoparticle hybrid material. J. Phys. Chem. C.

[29] Halder A., Zhang M., Chi Q. (2017). Electroactive and biocompatible functionalization of graphene for the development of biosensing platforms. Biosens. Bioelectron..

[30] Nigam S., Chandra S., Bahadur D. (2015). Dendrimers based electrochemical biosensors. J. Biomed. Res..

[31] Arotiba O., Owino J., Songa E., Hendricks N., Waryo T., Jahed N., Baker P., Iwuoha E. (2008). An electrochemical DNA biosensor developed on a nanocomposite platform of gold and poly (propyleneimine) dendrimer. Sensors.

[32] Arotiba O.A., Baker P.G., Mamba B.B., Iwuoha E.I. (2011). The application of electrodeposited poly (propylene imine) dendrimer as an immobilisation layer in a simple electrochemical DNA biosensor. Int. J. Electrochem. Sci..

[33] Castillo G., Spinella K., Poturnayová A., Šnejdárková M., Mosiello L., Hianik T. (2015). Detection of aflatoxin B1 by aptamer-based biosensor using PAMAM dendrimers as immobilization platform. Food Control.

[34] Hasanzadeh M., Shadjou N., Eskandani M., Soleymani J., Jafari F., de la Guardia M. (2014). Dendrimer-encapsulated and cored metal nanoparticles for electrochemical nanobiosensing. TrAC Trends Anal. Chem..

[35] Dervisevic M., Dervisevic E., Şenel M. (2018). Design of amperometric urea biosensor based on self-assembled monolayer of cystamine/PAMAM-grafted MWCNT/Urease. Sens. Actuators B-Chem..

[36] Idris A.O., Mabuba N., Arotiba O.A. (2018). A dendrimer supported electrochemical immunosensor for the detection of alpha-feto protein—A cancer biomarker. Electroanalysis.

[37] Tshikalaha P., Arotiba O.A. (2015). Dendrimer supported electrochemical immunosensor for the detection of cholera toxin in water. Int. J. Electrochem. Sci..

[38] Zhiguo G., Shuping Y., Zaijun L., Xiulan S., Guangli W., Yinjun F., Junkang L. (2011). An ultrasensitive electrochemical biosensor for glucose using CdTe-CdS core—Shell quantum dot as ultrafast electron transfer relay between graphene-gold nanocomposite and gold nanoparticle. Electrochim. Acta.

[39] Butwong N., Zhou L., Moore E., Srijaranai S., Luong J.H., Glennon J.D. (2014). A highly sensitive hydrogen peroxide biosensor based on hemoglobin immobilized on cadmium sulfide quantum dots/chitosan composite modified glassy carbon electrode. Electroanalysis.

[40] Zhang C., Lou J., Tu W., Bao J., Dai Z. (2015). Ultrasensitive electrochemical biosensing for DNA using quantum dots combined with restriction endonuclease. Analyst.

[41] Han E., Yang Y., He Z., Cai J., Zhang X., Dong X. (2015). Development of tyrosinase biosensor based on quantum dots/chitosan nanocomposite for detection of phenolic compounds. Anal. Biochem..

[42] Yerga D.M., García M.B., García A.C. (2014). Electrochemical immunosensor for anti-tissue transglutaminase antibodies based on the in situ detection of quantum dots. Talanta.

[43] Petryayeva E., Algar W.R. (2014). Multiplexed homogeneous assays of proteolytic activity using a smartphone and quantum dots. Anal. Chem..

[44] Yue Z., Lisdat F., Parak W.J., Hickey S.G., Tu L., Sabir N., Bigall N.C. (2013). Quantum-dot-based photoelectrochemical sensors for chemical and biological detection. ACS Appl. Mater. Interfaces.

[45] Chen J., Liu Y., Zhao G.-C. (2016). A novel photoelectrochemical biosensor for tyrosinase and thrombin detection. Sensors.

[46] Wang W., Bao L., Lei J., Tu W., Ju H. (2012). Visible light induced photoelectrochemical biosensing based on oxygen-sensitive quantum dots. Anal. Chim. Acta.

[47] Zheng M., Cui Y., Li X., Liu S., Tang Z. (2011). Photoelectrochemical sensing of glucose based on quantum dot and enzyme nanocomposites. J. Electroanal. Chem..

[48] Ncapayi V., Parani S., Songca S.P., Kodama T., Oluwafemi O.S. (2017). Simple green synthesis of amino acid functionalised CdTe/CdSe/ZnSe core-multi shell with improved cell viability for cellular imaging. Mater. Lett..

[49] Yu W., Qu L., Guo W., Peng X. (2003). Experimental determination of the extinction coefficient of Cd nanocrystal. Chem. Mater..

[50] Mohan S., Oluwafemi O.S., Songca S.P., Osibote O.A., George S.C., Kalarikkal N., Thomas S. (2014). Facile synthesis of transparent and fluorescent epoxy-CdSe-CdS-ZnS core-multi shell polymer nanocomposite. New J. Chem..

[51] Hai L., Nghia N., Nga P., Chinh V., Trang N., Hanh V. (2009). Preparation and spectroscopic investigation of colloidal CdSe/CdS/ZnS core/multishell nanostructure. J. Exp. Nanosci..

[52] Talapin D.V., Mekis I., Götzinger S., Kornowski A., Benson O., Weller H. (2004). CdSe/CdS/ZnS and CdSe/ZnSe/ZnS Core-Shell-Shell Nanocrystals. J. Phys. Chem. B.

[53] Wang Y., Liang X., Ma X., Hu Y., Hu X., Li X., Fan J. (2014). Simple and greener synthesis of highly photoluminescence Mn^2+^-doped ZnS quantum dots and its surface passivation mechanism. Appl. Surf. Sci..

[54] Jothi N.N., Joshi A.G., Vijay R.J., Muthuvinayagam A., Sagayaraj P. (2013). Investigation on one-pot hydrothermal synthesis, structural and optical properties of ZnS quantum dots. Mater. Chem. Phys..

[55] Dabbousi B.O., Rodriguez-Viejo J., Mikulec F.V., Heine J.R., Mattoussi H., Ober R., Jensen K.F., Bawendi M.G. (1997). (CdSe) ZnS core-shell quantum dots: Synthesis and characterization of a size series of highly luminescent nanocrystallites. J. Phys. Chem. B.

[56] Xu B., Cai B., Liu M., Fan H. (2013). Ultraviolet radiation synthesis of water dispersed CdTe/CdS/ZnS core–shell–shell quantum dots with high fluorescence strength and biocompatibility. Nanotechnology.

[57] Trindade T., O’Brien P., Pickett N.L. (2001). Nanocrystalline semiconductors: Synthesis, properties, and perspectives. Chem. Mater..

[58] Murray C., Sun S., Gaschler W., Doyle H., Betley T., Kagan C. (2001). Colloidal synthesis of nanocrystals and nanocrystal supperlattice. IBM J. Res. Dev..

[59] Smith A.M., Mohs A.M., Nie S. (2009). Tuning the optical and electronic properties of colloidal nanocrystals by lattice strain. Nat. Nanotechnol..

[60] Yan C., Tang F., Li L., Li H., Huang X., Chen D., Meng X., Ren J. (2010). Synthesis of aqueous CdTe/CdS/ZnS core/shell/shell quantum dots by a chemical aerosol flow method. Nanoscale Res. Lett..

[61] Gui R., An X. (2013). Layer-by-layer aqueous synthesis, characterization and fluorescence properties of type-II CdTe/CdS core/shell quantum dots with near-infrared emission. RSC Adv..

[62] Ndangili P.M., Arotiba O.A., Baker P.G., Iwuoha E.I. (2010). A potential masking approach in the detection of dopamine on 3-mercaptopropionic acid capped ZnSe quantum dots modified gold electrode in the presence of interferences. J. Electroanal. Chem..

[63] Arotiba O.A., Owino J.H., Baker P.G., Iwuoha E.I. (2010). Electrochemical impedimetry of electrodeposited poly (propylene imine) dendrimer monolayer. J. Electroanal. Chem..

[64] Volontè F., Pollegioni L., Molla G., Frattini L., Marinelli F., Piubelli L. (2010). Production of recombinant cholesterol oxidase containing covalently bound FAD in Escherichia coli. BMC Biotechnol..

[65] Ruecha N., Siangproh W., Chailapakul O. (2011). A fast and highly sensitive detection of cholesterol using polymer microfluidic devices and amperometric system. Talanta.

[66] Shrestha B.K., Ahmad R., Shrestha S., Park C.H., Kim C.S. (2017). In situ synthesis of cylindrical spongy polypyrrole doped protonated graphitic carbon nitride for cholesterol sensing application. Biosens. Bioelectron..

[67] Tripathy N., Ahmad R., Kim E.Y., Khang G., Hahn Y.B. (2014). Cholesterol biosensing based on highly immobilized ChOx on ZnO hollow nanospheres. RSC Adv..

[68] Ahmad R., Tripathy N., Kim S.H., Umar A., Al-Hajry A., Hahn Y.B. (2014). High performance cholesterol sensor based on ZnO nanotubes grown on Si/Ag electrodes. Electrochem. Commun..

[69] Ruecha N., Rangkupan R., Rodthongkum N., Chailapakul O. (2014). Novel paper-based cholesterol biosensor using graphene/polyvinylpyrrolidone/polyaniline nanocomposite. Biosens. Bioelectron..

[70] Giri A.K., Charan C., Saha A., Shahi V.K., Panda A.B. (2014). An amperometric cholesterol biosensor with excellent sensitivity and limit of detection based on an enzyme-immobilized microtubular ZnO@ZnS heterostructure. J. Mater. Chem. A.

[71] Sekretaryova A.N., Beni V., Eriksson M., Karyakin A.A., Turner A.P., Vagin M.Y. (2014). Cholesterol self-powered biosensor. Anal. Chem..

[72] Rahman M.M., Li X.-B., Kim J., Lim B.O., Ahammad A.S., Lee J.-J. (2014). A cholesterol biosensor based on a bi-enzyme immobilized on conducting poly (thionine) film. Sens. Actuators B.

